# *Wt1* Is Required for the Regression of Müllerian Ducts in Male Mice by Inducing *Wif1* and *Osx* Expression

**DOI:** 10.1111/cpr.70264

**Published:** 2026-07-14

**Authors:** Min Chen, Xin Qi, Shanshan Qin, Jia Kang, Changhuo Cen, Miao Guo, Yang Gao, Mengyue Wang, Jiayi Li, Xiuhong Cui, Yanbo Wang, Lan Zhu, Fei Gao

**Affiliations:** ^1^ State Key Laboratory of Organ Regeneration and Reconstruction, Institute of Zoology Chinese Academy of Sciences Beijing China; ^2^ Institute for Stem Cell and Regeneration Chinese Academy of Sciences Beijing China; ^3^ Beijing Institute for Stem Cell and Regenerative Medicine Beijing China; ^4^ University of Chinese Academy of Sciences Beijing China; ^5^ College of Veterinary Medicine, Inner Mongolia Agricultural University Key Laboratory of Clinical Diagnosis and Treatment Technology in Animal Disease, Ministry of Agriculture Hohhot China; ^6^ Department of Obstetrics and Gynecology, National Clinical Research Center for Obstetric and Gynecologic Diseases, Peking Union Medical College Hospital, State Key Laboratory for Complex Severe and Rare Diseases, Peking Union Medical College Chinese Academy of Medical Sciences Beijing China; ^7^ College of Life Sciences and Food Engineering Inner Mongolia Minzu University Tongliao Inner Mongolia China

**Keywords:** Müllerian ducts, *Osx*, regression, *Wif1*, *Wt1*

## Abstract

In mammals, Müllerian ducts (MDs) are the precursors of the female reproductive tract which regress in males during embryonic development. Failure of MD regression results in persistent Müllerian duct syndrome (PMDS) in humans. Although several factors essential for MD regression have been identified, the underlying regulatory network remains incompletely understood. In this study, we found that the Wilms tumour gene (*Wt1*) was highly expressed in the MD mesenchyme of male mice. Mesenchyme‐specific inactivation of *Wt1* resulted in MD retention and male infertility. The expression of *Amh* in the testes and its receptor *Amhr2* in the MD mesenchyme remained unchanged in *Wt1* ^*−/flox*^ *; Amhr2‐cre* male mice. Instead, the expression of Wnt inhibitory factor 1 (*Wif1*) and *Osterix* (*Osx*) was significantly reduced, accompanied by nuclear accumulation of β‐catenin in the MD mesenchyme of *Wt1*‐deficient mice. Further studies revealed that *Wif1* and *Osx* were direct transcriptional targets of WT1. These findings identify *Wt1* as a previously unrecognized mesenchymal regulator of MD regression by inducing *Wif1* and *Osx* expression and provide new insights into the regulatory mechanisms underlying MD regression as well as the aetiology of reproductive tract disorders associated with *WT1* mutations.

## Graphical abstract

*Wt1* expressed in the MD mesenchyme promotes MD regression by inducing *Wif1* and *Osx* transcription. In male mice, mesenchyme‐specific inactivation of *Wt1* results in MD retention.

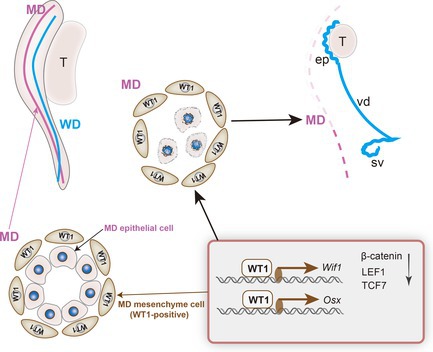

## 1 Introduction

In mammals, the Müllerian ducts (MDs) and Wolffian ducts (WD) are the precursors of the female and male reproductive tracts respectively, and both of them are located within the mesonephroi during early embryonic stage. Following sex differentiation, the Wolffian ducts regress in females, whereas the MDs develop into the female reproductive tract, including the oviduct, uterus, and upper vagina. In males, the MDs regress, while the Wolffian ducts persist and differentiate into the male reproductive tract, including the seminal vesicles, vas deferens, and epididymis [1, 2]. Regression of the MDs is triggered by anti‐Müllerian hormone (AMH) secreted from testicular Sertoli cells [3]. Previous studies have demonstrated that AMH induces MD regression by interacting with its type II serine/threonine kinase receptor, AMHR2, in the MD mesenchyme, which subsequently phosphorylates and activates the type I receptors ACVR1 and BMPR1A. These activated receptors then phosphorylate the receptor‐regulated SMADs (R‐SMADs)–SMAD1, SMAD5, and SMAD8, which in turn associate with SMAD4. This resulting complex translocates into the nucleus to regulate the transcription of downstream target genes [4, 5, 6].

In both *Amh* [7] and *Amhr2* [8] knockout mice, the MDs fail to regress in males and give rise to uterine and oviductal structures, coexisting with Wolffian duct‐derived male reproductive organs, such as seminal vesicles, vas deferens, and epididymis. In humans, mutations in *AMH* or *AMHR2* cause persistent Mullerian duct syndrome (PMDS), characterized by the retention of MD‐derived tissues, including the uterus and oviducts, in male patients. Most patients with PMDS are infertile because these retained tissues physically block sperm passage [9, 10]. In *Bmpr1a* knockout mouse model, approximately 50% of male mice retain MD derivatives [11], whereas inactivation of *Acvr1* does not affect the regression of MDs. However, all *Bmpr1a* and *Acvr1* double knockout mice display complete MD retention [6]. The functions of *Smad1*, *Smad5*, and *Smad8* are also redundant; triple conditional knockout males exhibit complete retention of the MDs, whereas loss of any single gene has no effect on MD regression [6]. Zinc finger transcription factor *Osterix* (*Osx*) is reported to functions downstream of AMH signalling, and *Osx* inactivation resulted in delayed MD regression in male mice [12].

The WNT/β‐catenin pathway has also been reported to act downstream of AMH signalling to mediate MD regression [13]. *Wnt7a* is involved in MD regression by regulating *Amhr2* expression in the mesenchyme, and male mice lacking *Wnt7a* fail to undergo MD regression [14]. Constitutive activation of β‐catenin in the MD mesenchyme results in focal MD retention in male mice [15]. Interestingly, inactivation of β‐catenin also impairs MD regression [13], suggesting that a finely tuned level of β‐catenin activity in the mesenchyme is essential for normal regression. Despite these findings, the molecular mechanisms underlying MD regression remain largely elusive.

The Wilms tumour gene (*Wt1*) encodes a transcription factor that is expressed in testicular Sertoli cells and ovarian granulosa cells, and is essential for the specification and maintenance of these supporting cell lineages [16, 17]. Conditional inactivation of *Wt1* in testicular Sertoli cells impairs normal MD regression in male mice, leading to the presence of a uterus in male mice. This phenotype results from a marked reduction in AMH secretion from Sertoli cells following *Wt1* inactivation [18]. In addition to the testis, *Wt1* is also expressed in the MD mesenchyme. However, whether *Wt1* is involved in MD development remains unclear. A previous study reported that *Wt1* acts as an activator of *Amhr2* by directly binding to the promoter region of *Amhr2* gene [19]. Nevertheless, this study was primarily performed using in vitro cultured cell lines, and whether *Wt1* is required for MD regression has not been determined. In the present study, we found that WT1 is also highly expressed in the MD mesenchyme during embryonic stage (Figure S1, E14.5‐E17.5). To determine the functions of *Wt1* in MD development, we generated *Wt1* ^*−/flox*^ *; Amhr2‐cre* mice, in which *Wt1* is specifically deleted in the MD mesenchyme. We found that *Wt1* ^*−/flox*^ *; Amhr2‐cre* males exhibited failure of MD regression and retained uterine structures. Notably, the expression of *Amh* in Sertoli cells and *Amhr2* in the MD mesenchyme were comparable to those in control males. Further analyses revealed that the expression of Wnt inhibitory factor 1, *Wif1*, was downregulated and β‐catenin signalling was activated in *Wt1*‐deficient MD mesenchyme. We also identified *Osx* as another WT1 target gene in the mesenchyme whose downregulation may further contribute to MD persistence.

## 2 Results

### 2.1 Müllerian Ducts Failed to Regress in *Wt1^−/flox^; Amhr2‐Cre* Male Mice

**FIGURE 1:**
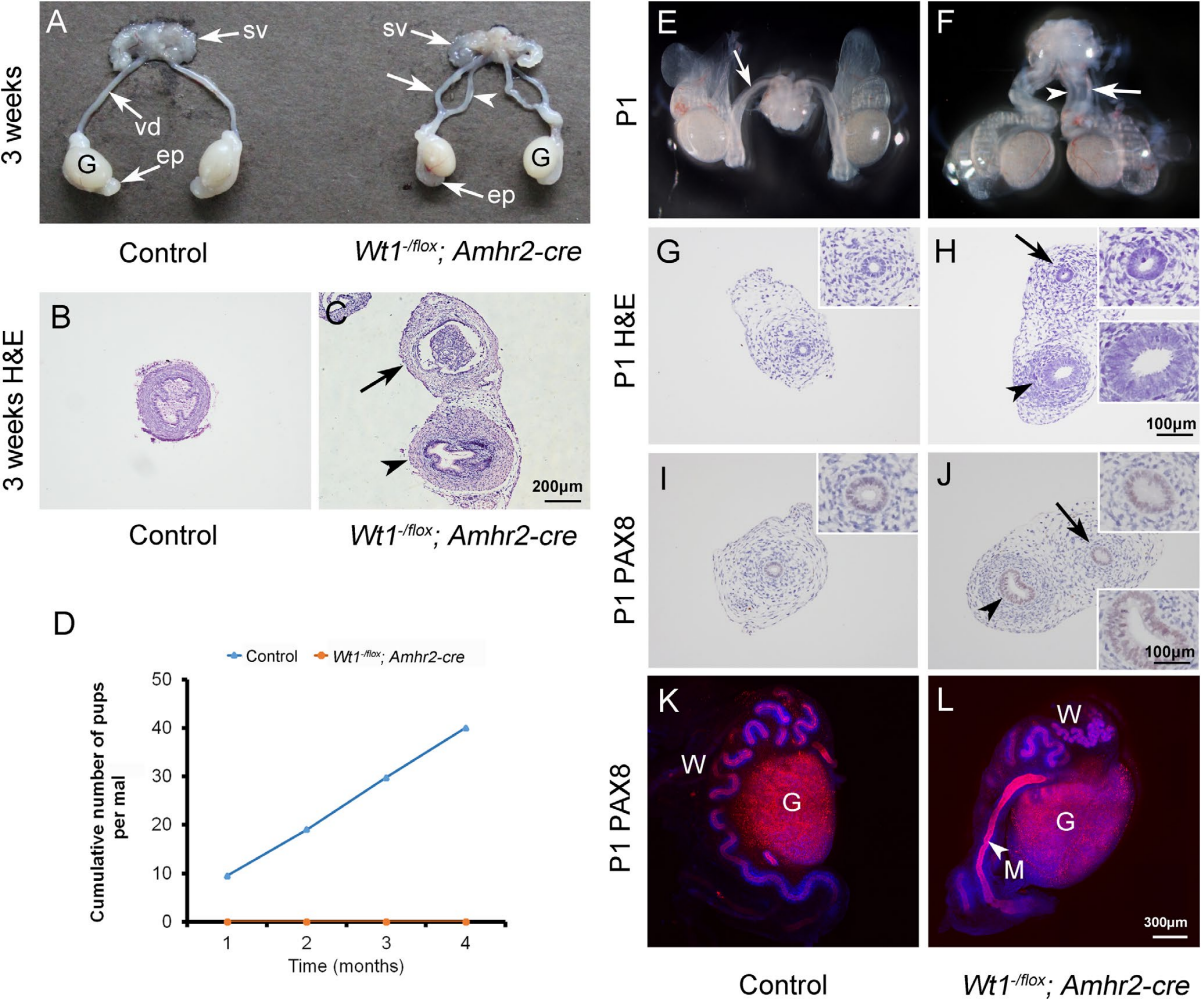
Müllerian ducts were retained in *Wt1* ^*−/flox*^ *; Amhr2‐cre* male mice. (A) Gross morphology of the reproductive system in control and *Wt1* ^*−/flox*^ *; Amhr2‐cre* male mice at 3 weeks of age. In addition to the seminal vesicle (sv), vas deferens (vd), and epididymis (ep), uterus (arrowhead) was observed in *Wt1* ^*−/flox*^ *; Amhr2‐cre* males. (B, C) H&E staining of the vas deferens in controls (B), and both the vas deferens and uterus in *Wt1* ^*−/flox*^ *; Amhr2‐cre* males (C, arrow and arrowhead). (D) Fertility test of control and *Wt1* ^*−/flox*^ *; Amhr2‐cre* male mice. The average cumulative number of pups produced per male over 4‐months is presented. (E, F) Gross morphology of the reproductive system in control (E) and *Wt1* ^*−/flox*^ *; Amhr2‐cre* males (F) at postnatal day 1 (P1). Only the vas deferens was observed in control males (E, arrow), whereas both the vas deferens and uterus were present in *Wt1* ^*−/flox*^ *; Amhr2‐cre* males (F, arrow and arrowhead). (G, H) H&E staining showed the vas deferens in control males (G) and both the vas deferens and uterus in *Wt1* ^*−/flox*^ *; Amhr2‐cre* males (H, arrow and arrowhead) at P1. (I, J) Immunohistochemical analysis of PAX8 showing staining in the vas deferens of control males (I) and in both the vas deferens and uterus in *Wt1* ^*−/flox*^ *; Amhr2‐cre* male mice (J, arrow and arrowhead) at P1. (K, L) Whole‐mount immunofluorescence staining for PAX8 at P1 confirmed Müllerian duct retention (arrowhead) in *Wt1* ^*−/flox*^ *; Amhr2‐cre* males. Nuclei were counterstained with DAPI (blue). M, Müllerian duct; W, Wolffian duct; G, gonad.

To specifically inactivate *Wt1* in the MD mesenchyme, *Wt1* ^*−/flox*^ *; Amhr2‐cre* mice were obtained by crossing *Wt1* ^*+/−*^ *; Amhr2‐cre* mice with *Wt1* ^*flox/flox*^ mice. The size and morphology of testes in *Wt1* ^*−/flox*^ *; Amhr2‐cre* mice were comparable to those in control littermates at 3 weeks of age. However, in addition to seminal vesicles, vas deferens, and epididymis, the uterus was also observed in *Wt1* ^*−/flox*^ *; Amhr2‐cre* males (Figure 1A, arrowheads). Haematoxylin and eosin (H&E) staining further confirmed that the MDs failed to regress in *Wt1* ^*−/flox*^ *; Amhr2‐cre* mice (Figure 1B,C). The results of the fertility test showed that *Wt1* ^*−/flox*^ *; Amhr2‐cre* males were completely infertile (Figure 1D). We further examined neonatal mice and found that the retention of MDs was already evident in *Wt1* ^*−/flox*^ *; Amhr2‐cre* males (Figure 1F, arrowheads). Paired box 8 (*Pax8*) is a transcription factor expressed in the epithelium of the Mullerian and Wolffian ducts as well as their derivatives. The results of both H&E (Figure 1G,H) and PAX8 (Figure 1I,J) staining showed that the vas deferens were present in control males, whereas *Wt1* ^*−/flox*^ *; Amhr2‐cre* males possessed both vas deferens and uterine structures. Whole‐mount immunofluorescence staining with anti‐PAX8 antibody further confirmed the persistence of MDs in *Wt1*‐deficient males but not in controls (Figure 1K,L).

We next examined the retention of MDs during embryonic stages using immunohistochemical analysis of PAX2 and PAX8. As shown in Figure S2, both MDs and WDs were present in control and *Wt1* ^*−/flox*^ *; Amhr2‐cre* male embryos at E15.5 (A, B, E, F). The MDs had nearly regressed in control embryos at E17.5, whereas they remained clearly visible in the mesonephroi of *Wt1*‐deficient males along with the Wolffian ducts (C, D, G, H).

### 2.2 The Expression of AMH and Its Receptors Was Not Affected in *Wt1*‐Deficient Males

**FIGURE 2:**
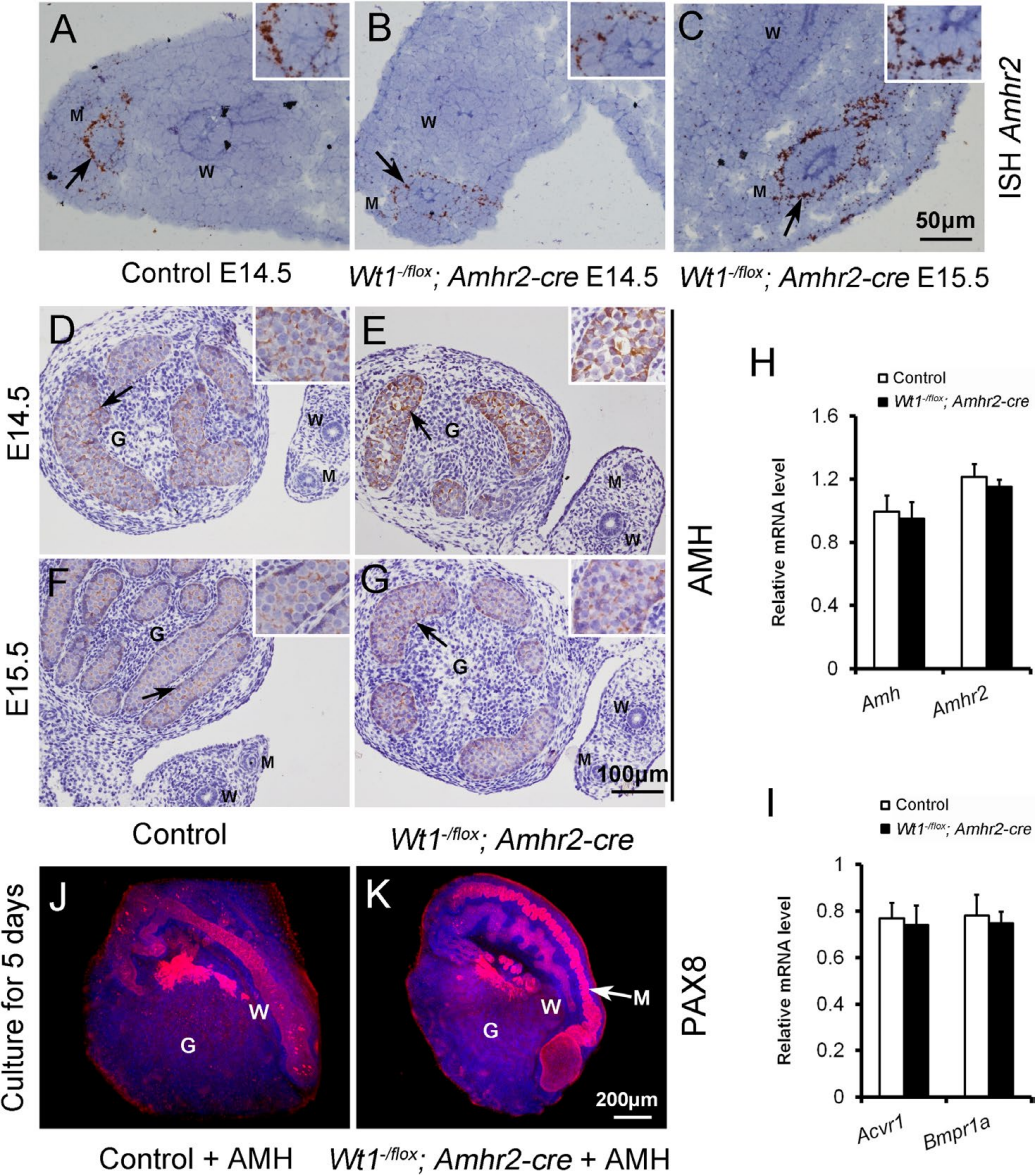
The persistence of Müllerian ducts in *Wt1* ^*−/flox*^ *; Amhr2‐cre* male mice was not due to altered expression of AMH or its receptors. (A–C) In situ hybridization showing *Amhr2* expression in Müllerian duct mesenchyme of control (A, arrow) and *Wt1* ^*−/flox*^ *; Amhr2‐cre* males (B, C, arrows). (D–G) Immunohistochemical analysis showing AMH expression in Sertoli cells of control (D, F, arrows) and *Wt1* ^*−/flox*^ *; Amhr2‐cre* males (E, G, arrows). (H, I) Quantitative RT‐PCR analysis of *Amh*, *Amhr2*, *Acvr1*, and *Bmpr1a* mRNA levels in Müllerian duct mesenchymal cells of control and *Wt1* ^*−/flox*^ *; Amhr2‐cre* males at E14.5. Data are presented as mean ± SEM. (J, K) Whole‐mount immunofluorescence of PAX8 in mesonephroi and testes. Mesonephroi and testes from control and *Wt1* ^*−/flox*^ *; Amhr2‐cre* males at E13.5 were cultured in vitro and treated with exogenous AMH protein for 5 days. Müllerian ducts in *Wt1* ^*−/flox*^ *; Amhr2‐cre* males (K, arrow) failed to regress, unlike those in controls (J). Nuclei were counterstained with DAPI (blue). M, Müllerian duct; W, Wolffian duct; G, gonad.

It has been reported that the expression of *Amhr2* is regulated by WT1 [19]. Therefore, we first examined *Amhr2* expression in the MD mesenchyme by in situ hybridization. As shown in Figure 2, *Amhr2* mRNA was detected in the MD mesenchyme of both control and *Wt1* ^*−/flox*^ *; Amhr2‐cre* mice, with no apparent difference in signal intensity between the two groups (Figure 2A–C, arrows). Real‐time PCR analysis further confirmed that the mRNA level of *Amhr2* was not altered in *Wt1* ^*−/flox*^ *; Amhr2‐cre* mice (Figure 2H). Moreover, the mRNA levels of the two type I AMH receptors, *Bmpr1a* and *Acvr1*, were also unchanged in *Wt1*‐deficient mesenchyme (Figure 2I).

To determine whether the failure of MD regression resulted from the defect of AMH secretion by Sertoli cells, the expression of MVH and SOX9 in the testes was examined by immunohistochemistry. As shown in Figure S3, normal structure of seminiferous tubules was observed in *Wt1* ^*−/flox*^ *; Amhr2‐cre* mice, and both the location and number of germ cells (Figure S3A,B) and Sertoli cells (Figure S3C,D) were comparable to those in control mice at postnatal day 1, when MD retention was already evident. AMH expression was examined by immunohistochemistry in the seminiferous tubules of both control and *Wt1* ^*−/flox*^ *; Amhr2‐cre* mice, and no obvious difference was observed between the two groups (Figure 2D–G). Consistently, real‐time PCR analysis confirmed that AMH expression in Sertoli cells was not changed in *Wt1* ^*−/flox*^ *; Amhr2‐cre* mice at E14.5 (Figure 2H). We also examined the expression of phosphorylated SMAD1/5/8 by immunohistochemistry. As shown in Figure S4, the expression of phosphorylated SMAD1/5/8 in the MD mesenchyme of *Wt1*‐deficient mice (Figure S4B,D) was comparable to that in controls (Figure S4A,C), indicating that mesenchymal *Wt1* deletion did not affect the activation of R‐SMADs.

To further exclude the possibility that AMH expression change contributes to the persistence of MD in *Wt1* ^*−/flox*^ *; Amhr2‐cre* males, mesonephros and gonads from control and *Wt1* ^*−/flox*^ *; Amhr2‐cre* embryos at E13.5 were cultured in vitro and treated with exogenous AMH. After five days of culture, MDs had almost completely regressed in the control group (Figure 2J), whereas MDs remained intact in the *Wt1*‐deficient group (Figure 2K). These results indicate that *Wt1* deletion mediated by *Amhr2‐cre* does not significantly affect the expression of AMH or its receptors, and that MD retention in *Wt1* ^*−/flox*^ *; Amhr2‐cre* males is not caused by altered expression of AMH or its receptors.

### 2.3 β‐Catenin Signalling Was Activated in *Wt1*‐Deficient MD Mesenchyme

**FIGURE 3:**
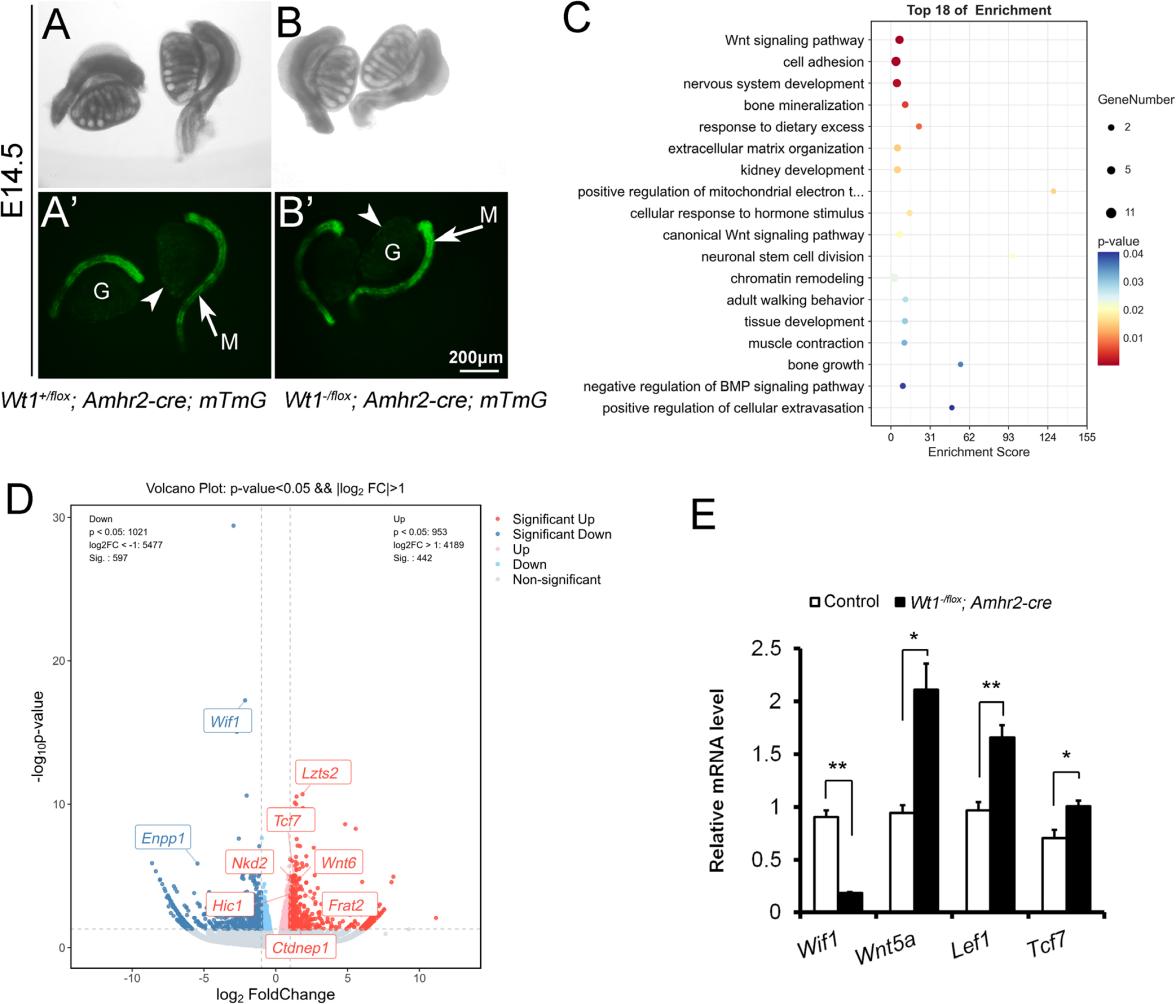
Wnt/β‐catenin signalling pathway was activated in Müllerian duct mesenchyme of *Wt1* ^*−/flox*^ *; Amhr2‐cre* male mice. A‐B. Bright‐field images of mesonephroi and testes from *Wt1* ^*+/flox*^ *; Amhr2‐cre; mTmG* (control, A) and *Wt1* ^*−/flox*^ *; Amhr2‐cre; mTmG* male (B) mice at E14.5. A', B′. Fluorescence images showing GFP expression in Müllerian ducts and testes from *Wt1* ^*+/flox*^ *; Amhr2‐cre; mTmG* (A') and *Wt1* ^*−/flox*^ *; Amhr2‐cre; mTmG* male (B′) mice at E14.5. AMHR2 is primarily expressed in the Müllerian duct mesenchyme (arrows), with very weak expression observed in Sertoli cells of the gonads (arrowheads). M, Müllerian duct; G, gonad. GFP‐positive mesenchymal cells of Müllerian ducts were isolated and sorted by FACS for RNA sequencing analysis. Pathway analysis was conducted using KEGG enrichment (C), and differentially expressed genes between *Wt1* ^*−/flox*^ *; Amhr2‐cre; mTmG* males and controls were visualized by volcano plot (D). (E) Quantitative RT‐PCR analysis of *Wif1*, *Wnt5a*, *Lef1*, and *Tcf7* mRNA levels in Müllerian duct mesenchymal cells of control and *Wt1* ^*−/flox*^ *; Amhr2‐cre* males at E14.5. Data are presented as mean ± SEM. *, *p* < 0.05. **, *p* < 0.01.

To explore the mechanisms underlying MD retention in *Wt1*‐deficient males, we used *mTmG* reporter mice to label *Amhr2‐*expressing MD mesenchymal cells. As shown in Figure 3, strong GFP signal was detected in the MD mesenchyme (A', B′, arrows) and very weak signals were observed in the testes (arrowheads). GFP‐positive mesenchymal cells from control and *Wt1*‐deficient mesonephroi were sorted by flow cytometry and subjected to RNA sequencing (Figure 3C,D, Figure S5A–D). Pathway analysis revealed that genes involved in *Wnt* signalling and cell adhesion were the most significantly enriched pathways between control and *Wt1*‐deficient cells (Figure 3C). Within the *Wnt* signalling pathway, *Wif1* was significantly downregulated, whereas *Tcf7* was upregulated in *Wt1*‐deficient mesenchyme (Figure 3D). These results were further confirmed by real‐time PCR analysis (Figure 3E). *Wif1* is an inhibitor of WNT/β‐catenin signalling pathway and knockdown of *Wif1* leads to up‐regulation of β‐catenin and its binding partners TCF7/LEF1 in the MD mesenchyme, which in turn causes the retention of MDs [20].

**FIGURE 4:**
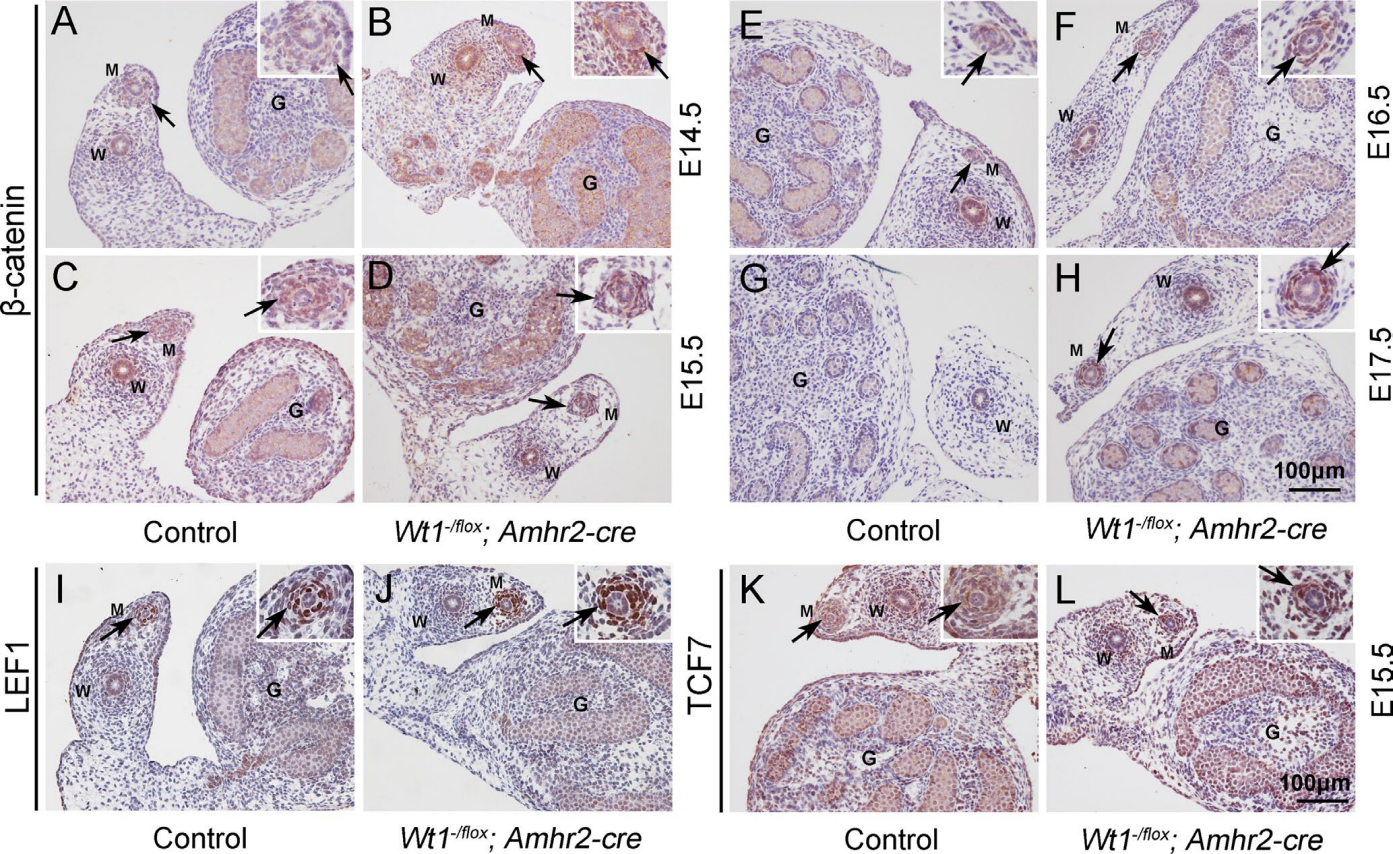
β‐catenin signalling was activated in Müllerian duct mesenchymal cells of *Wt1* ^*−/flox*^ *; Amhr2‐cre* male mice. The expressions of β‐catenin (A–H), LEF1 (I, J) and TCF7 (K, L) were examined by immunohistochemical analysis. In control male mice, β‐catenin was weakly expressed in both the cytoplasm and nucleus of Müllerian duct mesenchymal cells (A, C, E, G, arrows), whereas markedly stronger nuclear localization of β‐catenin protein was detected in *Wt1* ^*−/flox*^ *; Amhr2‐cre* males from E14.5 to E17.5 (B, D, F, H, arrows). Nuclear signals of the Wnt pathway effectors LEF1 (I, J, arrows) and TCF7 (K, L, arrows) were also elevated in Müllerian duct mesenchymal cells of *Wt1* ^*−/flox*^ *; Amhr2‐cre* males compared with controls at E15.5. M, Müllerian duct; W, Wolffian duct; G, gonad.

We next examined β‐catenin expression in the MD mesenchyme by immunohistochemical staining. β‐catenin was localized in MD mesenchyme of both control (Figure 4A,C,E, arrows) and *Wt1* ^*−/flox*^ *; Amhr2‐cre* males (Figure 4B,D,F,H, arrows). However, increased expression and prominent nuclear accumulation of β‐catenin protein were noted in *Wt1*‐deficient mesenchyme from E14.5 to E17.5 (Figure 4B,D,F,H, arrows). Consistently, the expression of β‐catenin binding partners LEF1 and TCF7 was also markedly elevated in the mesenchyme of *Wt1* ^*−/flox*^ *; Amhr2‐cre* males (Figure 4I–L, arrows), indicating β‐catenin signalling pathway was activated in *Wt1*‐deficient MD mesenchyme.

**FIGURE 5:**
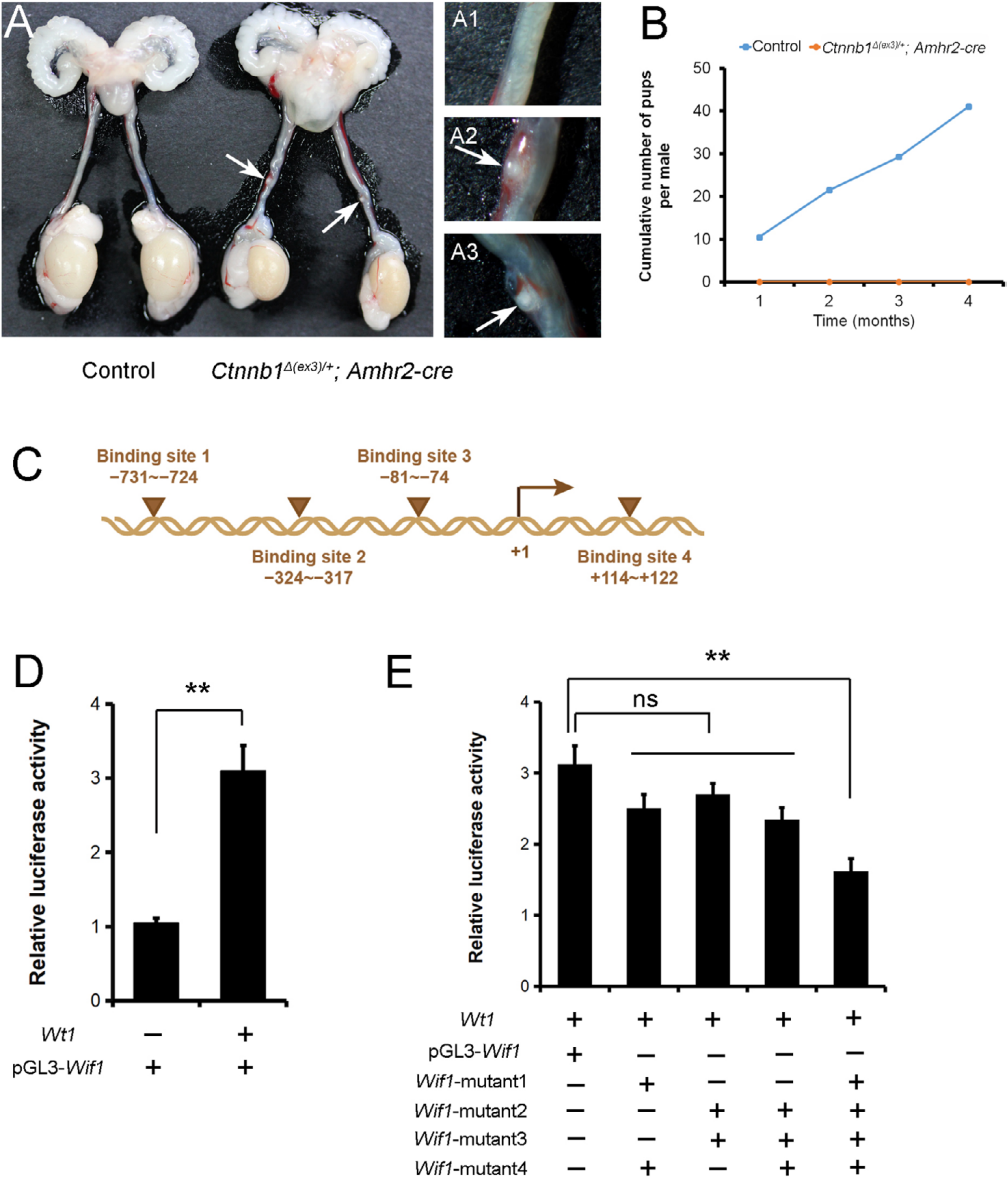
Activation of β‐catenin caused partial persistence of Müllerian ducts in male mice and *Wif1* expression was induced by WT1. (A) Activation of β‐catenin in the Müllerian duct mesenchyme led to partial retention of Müllerian ducts in adult males (arrows). A1 shows an enlarged view of the Müllerian duct in control mice, while A2 and A3 presented magnified views of the retained ducts in *Ctnnb1* ^*Δ(ex3)/+*^ *; Amhr2‐cre* mice from panel A. (B) Fertility test of male *Ctnnb1* ^*Δ(ex3)/+*^ *; Amhr2‐cre* mice. The mean cumulative number of pups per male was presented. (C) Schematic diagram of the predicted WT1‐binding sites within the promoter region of *Wif1* gene. (D) Luciferase reporter assay showing that *Wif1* promoter activity was significantly increased following transfection with a *Wt1*‐expressing vector. (E) Mutation of all four predicted WT1‐binding sites markedly reduced WT1‐induced luciferase activity of the *Wif1* promoter, whereas partial mutation of the four binding sites had no significant effect. In D and E, HEK293 cells were co‐transfected with either pCMS‐EGFP control or *Wt1*‐pCMS‐EGFP vectors together with wild‐type or mutated pGL3‐*Wif1* plasmids and harvested 48 h later for luciferase activity assays. Data are presented as mean ± SEM. **, *p* < 0.01; ns, not significant.

To investigate the functional consequences of β‐catenin activation in MD regression, we generated *Ctnnb1* ^*Δ(ex3)/+*^ *; Amhr2‐cre* mice, in which β‐catenin is constitutively activated by deletion of exon 3 in the MD mesenchyme [21]. No apparent defects in testis or Wolffian duct development were observed in adult *Ctnnb1* ^*Δ(ex3)/+*^ *; Amhr2‐cre* males. However, scattered foci of residual MD tissue were detected (Figure 5A, A2, A3, arrows), resulting in infertility probably due to obstruction of sperm transport within the reproductive tract (Figure 5B), which was consistent with previous reports [15].

We next examined whether the expression of *Wif1* is directly regulated by WT1. As shown in Figure 5C, four putative WT1‐binding sites were identified within the *Wif1* promoter region. Luciferase reporter assays showed that the luciferase activity of the *Wif1* promoter was significantly enhanced by WT1 (Figure 5D). Mutation of all four binding sites markedly attenuated WT1‐induced *Wif1* promoter activity, whereas mutation of two or three binding sites had no significant effect (Figure 5E).

### 2.4 *Osx* Expression Was Regulated by WT1 and Was Dramatically Reduced in the MD Mesenchyme of *Wt1^−/flox^; Amhr2‐Cre* Males

**FIGURE 6:**
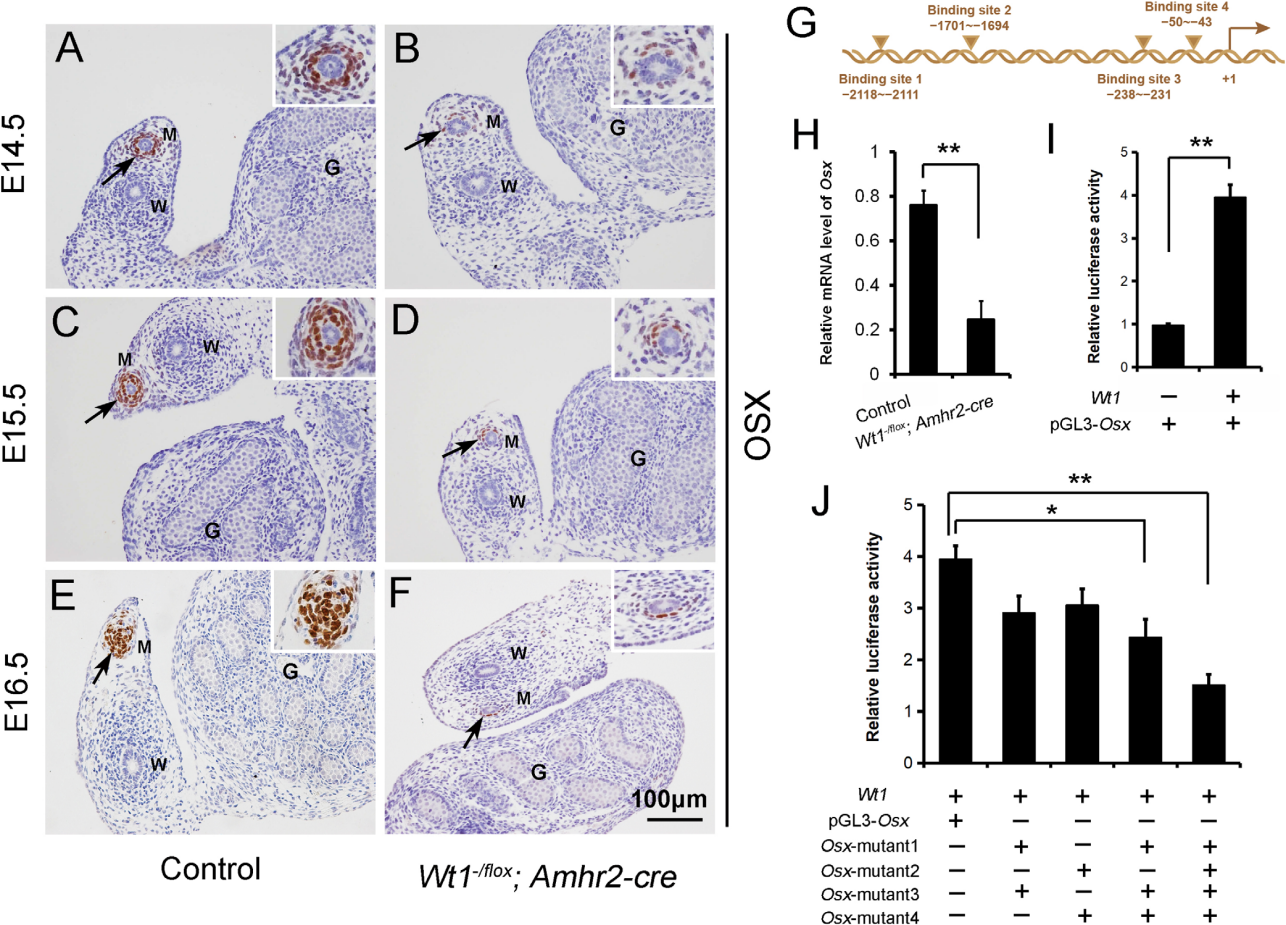
The expression of *Osx* was directly regulated by WT1. (A–F) OSX expression in the Müllerian duct mesenchyme was markedly reduced in *Wt1* ^*−/flox*^ *; Amhr2‐cre* male mice (B, D, F, arrows) compared with controls (A, C, E, arrows). Real‐time PCR analysis further confirmed a significant decrease in *Osx* mRNA levels in *Wt1* ^*−/flox*^ *; Amhr2‐cre* mice at E14.5 (H). (G) Schematic representation showing four predicted WT1‐binding sites within the *Osx* promoter region. (I) Luciferase activity driven by the *Osx* promoter was significantly increased following transfection with a *Wt1*‐expressing vector. (J) Mutation of three or all four WT1‐binding sites significantly reduced WT1‐induced *Osx* promoter activity. HEK293 cells were co‐transfected with either pCMS‐EGFP control or *Wt1*‐pCMS‐EGFP vectors together with wild‐type or mutant pGL3‐*Osx* reporter constructs, and luciferase activity was analysed 48 h post‐transfection. In (H–J), data are presented as mean ± SEM. *, *p* < 0.05; **, *p* < 0.01. M, Müllerian duct; W, Wolffian duct; G, gonad.

In addition to *Wif1*, the expression of transcription factor *Osx* was also markedly decreased in *Wt1* ^*−/flox*^ *; Amhr2‐cre* mice. During MD regression, *Osx* is specifically expressed in the MD mesenchyme of male embryos but not in females. Previous studies have reported that MD regression is delayed in *Osx*‐null male mice compared with control males [12]. Immunohistochemical analysis showed that high level of OSX protein was exclusively expressed in the MD mesenchyme of control males during embryonic stage, whereas its expression was dramatically reduced in *Wt1*‐deficient MD mesenchyme (Figure 6A–F). The reduction in *Osx* mRNA level in *Wt1*‐deficient mesonephroi was further confirmed by real‐time PCR analysis (Figure 6H). Sequence analysis identified four putative WT1‐binding sites within the *Osx* promoter region (Figure 6G). The promoter fragment was cloned into a pGL3‐basic luciferase reporter vector and co‐transfected with a *Wt1* expression plasmid. WT1 significantly increased the luciferase activity driven by *Osx* promoter (Figure 6I). This activation was markedly reduced when three or four binding sites were mutated, whereas mutation of two sites had no significant effect (Figure 6J). These results indicate that *Osx* is a direct downstream target of WT1 and that WT1‐induced MD regression is partially mediated through *Osx*.

## 3 Discussion

In mice, the MDs form between E11.5 and E13.5, extending caudally from the rostral mesonephros to the urogenital sinus. MD regression in male embryos begins at approximately E14.5 and completes by birth [22]. Although several genes involved in AMH‐induced MD regression have been identified [1, 2], the underlying regulatory mechanism is still not fully understood. The transcription factor *Wt1* is predominantly expressed in testicular Sertoli cells and the MD mesenchyme. While its roles in gonadal development are well established, its functions in the MD mesenchyme remain largely unexplored. In this study, we demonstrate that mesenchymal *Wt1* is essential for MD regression in male mice. Conditional deletion of *Wt1* in the mesenchyme led to failure of MD regression, resulting in the presence of a uterus in male mice in addition to Wolffian duct derivatives.

It is well established that MD regression is triggered by AMH secreted from Sertoli cells [3] via interaction with its receptors in the MD mesenchyme [4, 5, 6]. While *Amrhr2* is expressed predominantly in the MD mesenchyme, it is also weakly detected in testicular Sertoli cells. Our previous study also finds that inactivation of *Wt1* in Sertoli cells causes downregulation of AMH which in turn leads to MD retention [18]. In the present study, however, we found that testes development in mutant males was roughly normal, and both mRNA and protein levels of *Amh* were comparable to those in control males. Most importantly, MD regression in *Wt1* ^*−/flox*^ *; Amhr2‐cre* males could not be induced by exogenous AMH used in in vitro organ culture, indicating that the failure of MD regression is not due to defective AMH secretion. Although *Amhr2* has been reported as a downstream target of WT1, with its expression induced by WT1 [19], our results of in situ hybridization and quantitative PCR analyses showed that the expression of *Amhr2*, as well as the type I receptors *Bmpr1a* and *Acvr1* was unchanged in the MD mesenchyme of *Wt1* ^*−/flox*^ *; Amhr2‐cre* males. Moreover, the activation of R‐SMADs downstream of AMH receptor signalling was not affected. These findings indicate that the failure of MD regression in *Wt1* mutant mice is not caused by altered expression of AMH receptors or impaired R‐SMAD activation.

*Wif1*, a secreted frizzled‐related protein, inhibits the Wnt signalling by binding to Wnt ligands and preventing their interaction with receptors [23]. *Wif1* is specifically expressed in the MD mesenchyme in male but not in female mice during MD regression, and its expression can be induced by AMH [20]. Knockdown of *Wif1* by siRNA increases nuclear accumulation of β‐catenin protein and upregulates LEF1 and TCF7 in MD mesenchyme, leading to MD retention in organ culture assays [20]. However, no MD‐derived tissues are observed in *Wif1* knockout mice, suggesting other functional redundant factors are probably involved in this process [20]. The Wnt/β‐catenin and AMH/AMHR2 pathways interact to regulate MD regression, and β‐catenin serves as a central effector of canonical WNT signalling that modulates partial AMH/AMHR2 activity [2]. Our results show that constitutive activation of β‐catenin in the MD mesenchyme caused partial MD retention, which is consistent with previous studies [15]. Conversely, inactivation of β‐catenin also impairs MD regression in male mice [13], indicating that a balanced β‐catenin activity is essential for proper MD regression. In our study, *Wif1* expression was markedly decreased in the MD mesenchyme of *Wt1*‐deficient males, whereas β‐catenin and its transcriptional partners LEF1 and TCF7 were increased. Further analysis confirmed that the expression of *Wif1* is directly regulated by WT1. These results suggest that MD retention in *Wt1* ^*−/flox*^ *; Amhr2‐cre* males is partially due to downregulation of *Wif1*, which in turn induces aberrant activation of β‐catenin signalling.

The *Osx* gene encodes a zinc finger transcription factor originally identified as a key regulator of osteoblast differentiation. *Osx* is also expressed in the MD mesenchyme in a male‐specific pattern, and *Osx*‐null males show delayed MD regression [12]. In this study, we found that *Osx* is another downstream target of WT1, and its expression was substantially reduced in *Wt1*‐deficient MD mesenchyme. Based on these results, we speculate that WT1 serves as a critical upstream regulator of MD regression by coordinating both Wnt/β‐catenin signalling and *Osx* activity. These two pathways probably act synergistically to regulate MD regression, which may explain why MD regression is not completely blocked in either β‐catenin‐activated or *Osx*‐null mice. As a Wnt inhibitory factor, *Wif1* can inhibit not only canonical Wnt/β‐catenin signalling but also noncanonical Wnt pathways [24]. Although the involvement of noncanonical Wnt pathways in MD regression has not been reported, it remains unclear whether pathways such as Wnt/Ca^2+^ or planar cell polarity (PCP) signalling are suppressed by *Wif1* and contribute to WT1‐mediated MD regression. This possibility warrants further investigation.

In summary, our findings demonstrate that mesenchymal *Wt1* is indispensable for MD regression by inducing *Wif1* and *Osx* expression (Figure S6). *WT1* mutations in humans are associated with developmental syndromes such as Denys–Drash syndrome (DDS) and Frasier syndrome. DDS is characterized by ambiguous or female external genitalia, dysgenetic gonads, and Wilms' tumour, whereas male patients with Frasier syndrome present with female external genitalia, streak gonads with gonadoblastoma, and progressive glomerulopathy [25, 26]. Importantly, both syndromes exhibit failed MD regression in affected males. Previous mechanistic studies have largely focused on the role of *Wt1* in testis development. Our findings uncover a pivotal function of *Wt1* in the MD mesenchyme during MD regression, which may represent an additional mechanism contributing to persistent MDs in these patients. These insights enhance our understanding of the aetiology and molecular basis of sex development disorders associated with *WT1* mutations.

## 4 Materials and Methods

### 4.1 Mice

All mouse experiments were conducted in accordance with protocols approved by the Institutional Animal Care and Use Committee (IACUC) of the Institute of Zoology, Chinese Academy of Sciences (IOZ‐IACUC‐2021‐039). Mice were maintained in a C57BL/6;129/SvEv mixed background. *Wt1* ^*−/flox*^ mice were crossed with mice carrying the *Wt1*‐null allele (*Wt1* ^*+/−*^) and with *Amhr2‐cre* transgenic mice. *Wt1* ^*+/flox*^ and *Wt1* ^*−/flox*^ males were used as controls. *Wt1* ^*−/flox*^ *; Amhr2‐cre; mTmG* mice were generated by crossing *Wt1* ^*+/−*^ *; Amhr2‐cre* males with *Wt1* ^*flox/flox*^ *; mTmG* females, and *Wt1* ^*+/flox*^ *; Amhr2‐cre; mTmG* males served as controls. *Ctnnb1* ^*Δ(ex3)/+*^ *; Amhr2‐cre* mice were produced by crossing *Ctnnb1* ^*Δ(ex3)/+*^males with *Amhr2‐cre* transgenic females. Genotyping was performed by PCR using DNA extracted from fetal tissues.

For fertility assays, four *Wt1* ^*+/flox*^ *; Amhr2‐cre* males, four *Ctnnb1* ^*Δ(ex3)/+*^ *; Amhr2‐cre* males, and four control males were each paired with four wild‐type females at 8 weeks of age. Each male–female pair was housed together for 4 months, and the number of pups produced during this period was recorded.

### 4.2 Plasmids, Transient Transfection, and Luciferase Assay

The *Wif1* promoter fragment was cloned into the SacI and BglII sites of the pGL3‐basic luciferase reporter vector using the primers ATAAGAGCTCTACGCGTCCAAGTCCGGCTTCT and GGAAGATCTTCCGAGCCATGGTGCTCAGGACC. The*Osx* promoter was inserted into the KpnI and NheI sites of the pGL3‐basic vector using the primers CGGGGTACCTCATTGGGATTGCATGCCACAG and CTAGCTAGCCAAGCAGAGAGGACGCCATCCTC. Mutant *Wif1* and *Osx* promoter constructs containing substitutions in WT1‐binding sites were generated by site‐directed mutagenesis using KOD‐Plus‐Neo Polymerase (TOYOBO, KOD‐401).

293 T cells were transfected with either wild‐type or mutant *Wif1* or *Osx* promoter luciferase reporters, the pRL‐TK Renilla luciferase plasmid, and either pCMS‐EGFP or *Wt1*‐pCMS‐EGFP using Lipofectamine 3000 (Invitrogen, L3000015). After 48 h, cells were lysed, and luciferase activity was measured with the Dual Luciferase Reporter Assay System (Promega, E1960).

### 4.3 Histological, Immunohistochemical, and in Situ Hybridization Analyses

Histological and immunohistochemical analyses were performed as previously described [27]. Briefly, embryonic mesonephroi and testes were dissected immediately after euthanasia, fixed in 4% paraformaldehyde, dehydrated through an ethanol gradient, and embedded in paraffin. For H&E staining, 5‐μm sections were incubated with haematoxylin to stain the nuclei, followed by eosin to stain cytoplasm. For immunohistochemistry, sections were blocked with 5% BSA, incubated with primary antibodies for 1 h, and then with the corresponding secondary antibodies for 1 h. The primary antibodies used were WT1 (abcam, ab89901), PAX8 (CST, 59019S), AMH (Santa Cruz, sc‐6886), MVH (Abcam, ab13840), SOX9 (Millipore, AB5535), β‐catenin (Abcam, ab6302), OSX (Abcam, ab209484), LEF1 (Abcam, ab137872), TCF7 (Proteintech, 14,464–1‐AP), PAX2 (Abcam, ab79389), and SMAD1/5/8 (phospho S463 + S465 + S467, Abcam, ab92698). Signal was visualized using a diaminobenzidine substrate kit, counterstained with haematoxylin, and examined under a Nikon microscope. Images were captured using a Nikon DS‐Ri1 CCD camera.

In situ hybridization was performed using the RNAscope 2.5 HD Brown assay kit (ACD, 322300) according to the manufacturer's instructions. In brief, paraffin‐embedded tissue sections are baked, deparaffinized, and subjected to pretreatment including hydrogen peroxide quenching, target retrieval in heated buffer, and protease digestion to expose target RNA. After applying a hydrophobic barrier, sections underwent probe hybridization using the mouse *Amhr2* probe (ACD, 489821), followed by sequential signal amplification steps (Amp 1–6), and finally chromogenic detection with DAB. Sections were counterstained with haematoxylin, dehydrated, cleared, and mounted for imaging with a Nikon DS‐Ri1 CCD camera.

### 4.4 Mesonephros and Testis Culture and Whole‐Mount Staining

Mesonephroi and testes were dissected from E13.5 embryos following euthanasia of pregnant mice. After genotyping, tissues from control and *Wt1* ^*−/flox*^ *; Amhr2‐cre* embryos were cultured on 0.4 μm pore‐size inserts (Millipore, PICM01250) placed in 24‐well plates containing DMEM/F12 supplemented with 10% FBS, with or without 5 μg/mL recombinant AMH (Cloud‐Clone, APA228Hu01). After 5 days of culture, mesonephros and testes were fixed in 4% paraformaldehyde, blocked in 2% BSA and 2% Triton X‐100 for 24 h at 4°C, incubated with anti‐PAX8 antibody for 48 h, and then with Cy3‐conjugated donkey anti‐rabbit IgG (Jackson ImmunoResearch, 711–165‐152) for 24 h. Following DAPI staining, tissues were cleared in the water‐based reagent SCALEVIEW‐A2 (FUJIFILM, 193–18,455) for 3 days and imaged with a high‐speed confocal microscope (Andor Dragonfly 200).

### 4.5 Micro‐Transcriptome Sequencing

Mesenchymal cells were isolated and sorted from ten *Wt1* ^*+/flox*^ *; Amhr2‐cre; mTmG* (Control) embryos and ten *Wt1* ^*−/flox*^ *; Amhr2‐cre; mTmG* embryos. For each genotype, cells from five embryos were pooled into a single sample, yielding two independent biological replicates per genotype. RNA‐seq analysis was subsequently performed on these samples by ANOROAD Genome. Cell lysates containing lysis buffer and RNase inhibitors were used as templates for reverse transcription with oligo(dT) primers to generate first‐strand cDNA. The resulting cDNA was subsequently amplified by PCR, and the amplified products were purified for library preparation. Library construction included DNA fragmentation, end repair, A‐tailing, adapter ligation, PCR enrichment, and quality control. The final libraries were sequenced on an Illumina platform using a paired‐end 150 bp (PE150) strategy. Raw sequencing reads obtained from the HiSeq platform were filtered to remove adapter contamination and low‐quality sequences, and the clean reads were aligned to the mouse reference genome using HISAT2. Gene expression levels were quantified and normalized as FPKM (Fragments Per Kilobase of transcript per Million mapped reads). Differential gene expression analysis was performed using DESeq2 based on raw count data from samples with biological replicates. Differentially expressed genes (DEGs) were defined using the following thresholds: |log2FoldChange| ≥ 1 and an adjusted *p* value (FDR, Benjamini‐Hochberg correction) < 0.05. The raw transcriptome sequencing data have been deposited in the SRA database under accession number PRJNA1367207.

### 4.6 Real‐Time RT‐PCR

Total RNA from MD mesenchyme cells was isolated using the RNeasy Kit (Aidlab, RN28) according to the manufacturer's instructions. First‐strand cDNAs were synthesized from 800 ng of total RNA using the reverse transcription kit (Aidlab, PC5801) and subsequently diluted for real‐time PCR using a SYBR Green Master Mix (Aidlab, PC3301). *Gapdh* was used as the endogenous control, and gene expression levels were quantified relative to *Gapdh* using the 2^‐ΔΔCT^ method. Primer sequences used for real‐time RT‐PCR are listed in Table S1.

### 4.7 Statistical Analysis

For immunohistochemical analyses, three to five mice per genotype at each time point were examined. Representative images shown reflect consistent findings observed across three to five mice for each genotype at each time point. For real‐time RT‐PCR, mesenchymal cells collected from three embryos were pooled to generate one biological sample, and three biological samples per genotype were analysed. Luciferase assays were performed at least three times using independent cell preparations. Quantitative data are presented as the mean ± SEM. Statistical analyses were conducted using GraphPad Prism version 9.0.0. Comparisons between two groups were performed using unpaired two‐tailed Student's *t*‐tests, whereas one‐way ANOVA was applied for analyses involving three or more groups. *p* < 0.05 was considered statistically significant.

## Author Contributions

**Min Chen:** funding acquisition, project administration, writing – original draft, review and editing. **Xin Qi, Shanshan Qin:** methodology, data curation. **Jia Kang:** conceptualization, methodology. **Changhuo Cen, Miao Guo, Yang Gao, Mengyue Wang, Jiayi Li, Xiuhong Cui, Yanbo Wang:** writing – review and editing. **Lan Zhu:** project administration, writing – review and editing. **Fei Gao:** conceptualization, funding acquisition, project administration, writing – original draft, review and editing.

## Funding

This work was supported by the Strategic Priority Research Program of the Chinese Academy of Sciences, XDB0820000, National Natural Science Foundation of China, 32530031, 82571907, 82421003, 32270902, 32360182, Initiative Scientific Research Program, Institute of Zoology, Chinese Academy of Sciences, 2023IOZ0102, and Central Government Guiding Funds for Local Science and Technology Development, 2023ZY0015.

## Conflicts of Interest

The authors declare no conflicts of interest.

## Supporting information


**Figure S1:** WT1 was highly expressed in the Müllerian duct mesenchyme. WT1 expression was examined by immunohistochemistry from E14.5 to E17.5. In the testis, WT1 was specifically localized to Sertoli cells. In the mesonephroi, high level of WT1 expression was also detected in the mesenchyme of Müllerian ducts (arrows). M, Müllerian duct; W, Wolffian duct; G, gonad.
**Figure S2:** Persistent Müllerian ducts were observed in *Wt1* ^*−/flox*^ *; Amhr2‐cre* male mice during embryonic stages. The expression of PAX2 (A‐D, arrows) and PAX8 (E‐H, arrows) was analysed by immunohistochemical analysis in control (A, C, E, G) and *Wt1* ^*−/flox*^ *; Amhr2‐cre* (B, D, F, H) male mice at E15.5 and E17.5. M, Müllerian duct; W, Wolffian duct; G, gonad.
**Figure S3:** The structure of seminiferous tubules was intact in *Wt1* ^*−/flox*^ *; Amhr2‐cre* male mice. The expression of MVH (A‐B, arrows) and SOX9 (C‐D, arrows) was examined by immunohistochemical staining in testis sections from control and *Wt1* ^*−/flox*^ *; Amhr2‐cre* male mice at postnatal day 1.
**Figure S4:** Activation of SMAD1/5/8 was unchanged in the Müllerian duct mesenchyme of *Wt1* ^*−/flox*^ *; Amhr2‐cre* male mice. The expression of phosphorylated SMAD1/5/8 (arrows) in Müllerian duct mesenchyme from control and *Wt1* ^*−/flox*^ *; Amhr2‐cre* male mice at E14.5 and E15.5 was examined by immunohistochemical staining.
**Figure S5:** RNA‐seq analysis of GFP‐positive Müllerian duct mesenchymal cells from control and *Wt1* ^*−/flox*^ *; Amhr2‐cre; mTmG* males. A. Heatmap of differentially expressed genes between *Wt1* ^*+/flox*^ *; Amhr2‐cre; mTmG* (Control) and *Wt1* ^*−/flox*^ *; Amhr2‐cre; mTmG* (CKO) male mice at E14.5. B‐C. Pearson correlation analysis of transcriptomes for control (B, green dots) and *Wt1* ^*−/flox*^ *; Amhr2‐cre; mTmG* groups (C, blue dots). Each dot represents a single gene. Red line, linear best fit; pink band, 95% confidence interval. The high R^2^ values (Control: 0.9233; CKO: 0.9538; *p* < 0.0001) confirm robust reproducibility within groups. D. Sample correlation heatmap. Red and larger circles indicate higher correlation; blue and smaller circles indicate lower correlation.
**Figure S6:** Schematic representation of mesenchymal WT1‐medicated Müllerian duct regression in male mice. During early embryonic development, the Müllerian duct (MD) and Wolffian duct (WD) are both present within the mesonephroi of male and female mice. After sex determination, the MD regresses in males, whereas the WD differentiates into the male reproductive tract, including the seminal vesicles (sv), vas deferens (vd), and epididymis (ep). MD regression is initiated by AMH secreted from Sertoli cells of the testis (T), which acts through its receptors in the MD mesenchyme. *Wt1* expressed in the MD mesenchyme promotes MD regression by inducing *Wif1* transcription, thereby suppressing β‐catenin signalling and reducing the expression of its binding partners LEF1 and TCF7. In addition, *Osx* is another WT1 target gene in the mesenchyme whose upregulation may further contribute to MD regression. Mesenchyme‐specific inactivation of *Wt1* results in MD retention in male mice without markedly affecting the expression of AMH or its receptors.
**Table S1:** Primers for real‐time PCR.

## Data Availability

The data that support the findings of this study are openly available in SRA database at https://www.ncbi.nlm.nih.gov/sra/PRJNA1367207, reference number PRJNA1367207.
