# Supplementary material for: Wt1 Is Required for the Regression of Müllerian Ducts in Male Mice by Inducing Wif1 and Osx Expression
Source: Cell Prolif. 2026 Jul 14:e70264. Online ahead of print. doi: 10.1111/cpr.70264 (PMC13366097; doi:10.1111/cpr.70264)
Supplement: Supplementary file 1 — Figure S1: WT1 was highly expressed in the Müllerian duct mesenchyme. WT1 expression was examined by immunohistochemistry from E14.5 to E17.5. In the testis, WT1 was specifically localized to Sertoli cells. In the mesonephroi, high level of WT1 expression was also detected in the mesenchyme of Müllerian ducts (arrows). M, Müllerian duct; W, Wolffian duct; G, gonad. Figure S2: Persistent Müllerian ducts were observed in Wt1 −/flox ; Amhr2‐cre male mice during embryonic stages. The expression of PAX2 (A‐D, arrows) and PAX8 (E‐H, arrows) was analysed by immunohistochemical analysis in control (A, C, E, G) and Wt1 −/flox ; Amhr2‐cre (B, D, F, H) male mice at E15.5 and E17.5. M, Müllerian duct; W, Wolffian duct; G, gonad. Figure S3: The structure of seminiferous tubules was intact in Wt1 −/flox ; Amhr2‐cre male mice. The expression of MVH (A‐B, arrows) and SOX9 (C‐D, arrows) was examined by immunohistochemical staining in testis sections from control and Wt1 −/flox ; Amhr2‐cre male mice at postnatal day 1. Figure S4: Activation of SMAD1/5/8 was unchanged in the Müllerian duct mesenchyme of Wt1 −/flox ; Amhr2‐cre male mice. The expression of phosphorylated SMAD1/5/8 (arrows) in Müllerian duct mesenchyme from control and Wt1 −/flox ; Amhr2‐cre male mice at E14.5 and E15.5 was examined by immunohistochemical staining. Figure S5: RNA‐seq analysis of GFP‐positive Müllerian duct mesenchymal cells from control and Wt1 −/flox ; Amhr2‐cre; mTmG males. A. Heatmap of differentially expressed genes between Wt1 +/flox ; Amhr2‐cre; mTmG (Control) and Wt1 −/flox ; Amhr2‐cre; mTmG (CKO) male mice at E14.5. B‐C. Pearson correlation analysis of transcriptomes for control (B, green dots) and Wt1 −/flox ; Amhr2‐cre; mTmG groups (C, blue dots). Each dot represents a single gene. Red line, linear best fit; pink band, 95% confidence interval. The high R2 values (Control: 0.9233; CKO: 0.9538; p < 0.0001) confirm robust reproducibility within groups. D. Sample correlation heatmap. Red and larger [file CPR-9999-e70264-s001.docx]

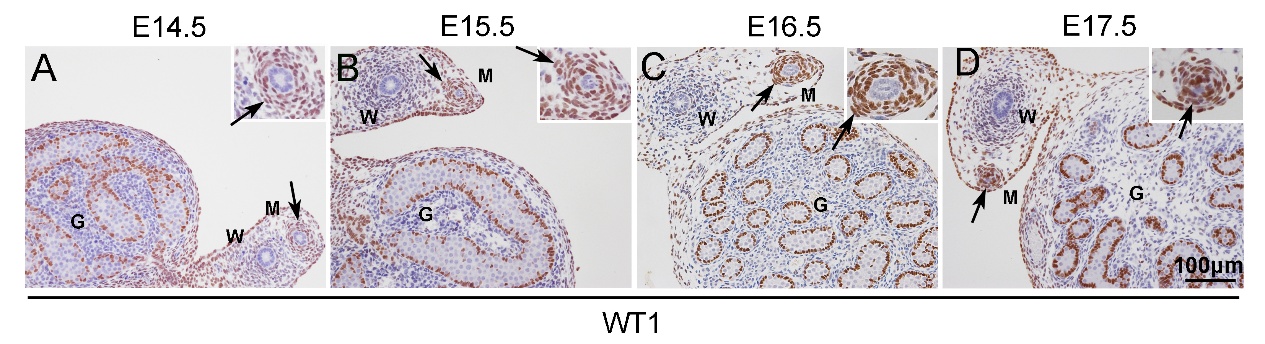


**Fig. S1. WT1 was abundantly expressed in the mesenchyme of Müllerian ducts.** WT1 expression was examined by immunohistochemistry from E14.5 to E17.5. In the testis, WT1 was specifically localized to Sertoli cells. In the mesonephroi, high level of WT1 was also detected in the mesenchyme of Müllerian ducts (arrows). M, Müllerian duct; W, Wolffian duct; G, gonad.


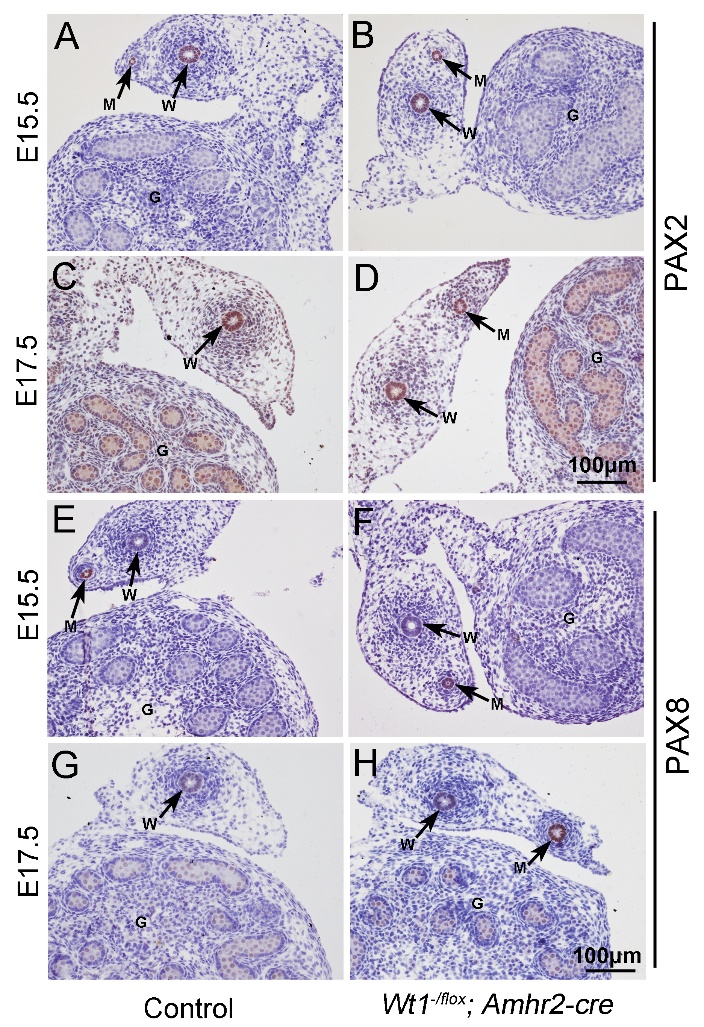


**Fig. S2. Persistent Müllerian ducts were observed in *Wt1^-/flox^; Amhr2-cre* male mice during embryonic stages.** The expression of PAX2 (A-D, arrows) and PAX8 (E-H, arrows) in control (A, C, E, G) and *Wt1^-/flox^; Amhr2-cre* (B, D, F, H) male mice at E15.5 and E17.5 was analyzed by immunohistochemical analysis. M, Müllerian duct; W, Wolffian duct; G, gonad.


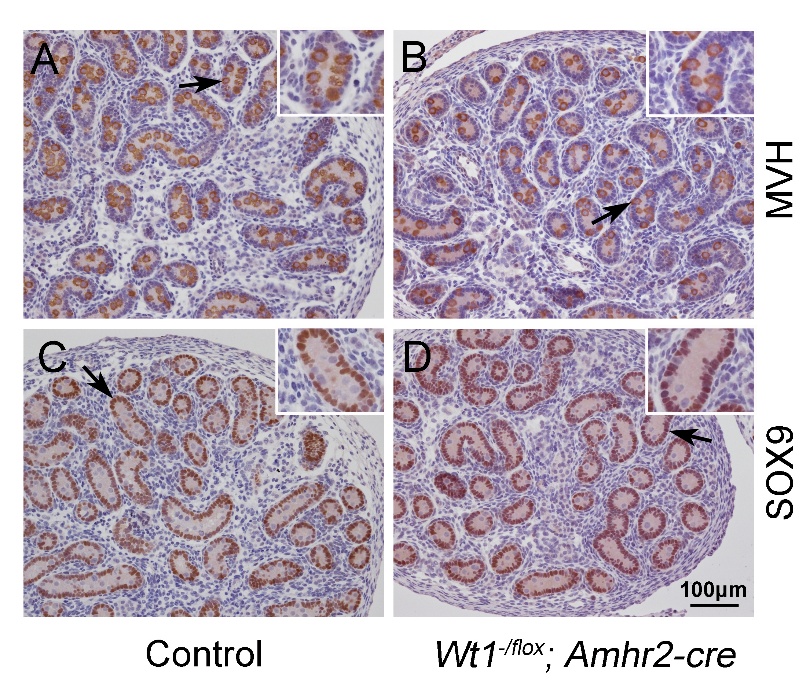


**Fig. S3. The structure of seminiferous tubules was intact in *Wt1^-/flox^; Amhr2-cre* male mice.** The expression of MVH (A-B, arrows) and SOX9 (C-D, arrows) in testis sections from control and *Wt1^-/flox^; Amhr2-cre* male mice at postnatal day 1 was examined by immunohistochemical staining.


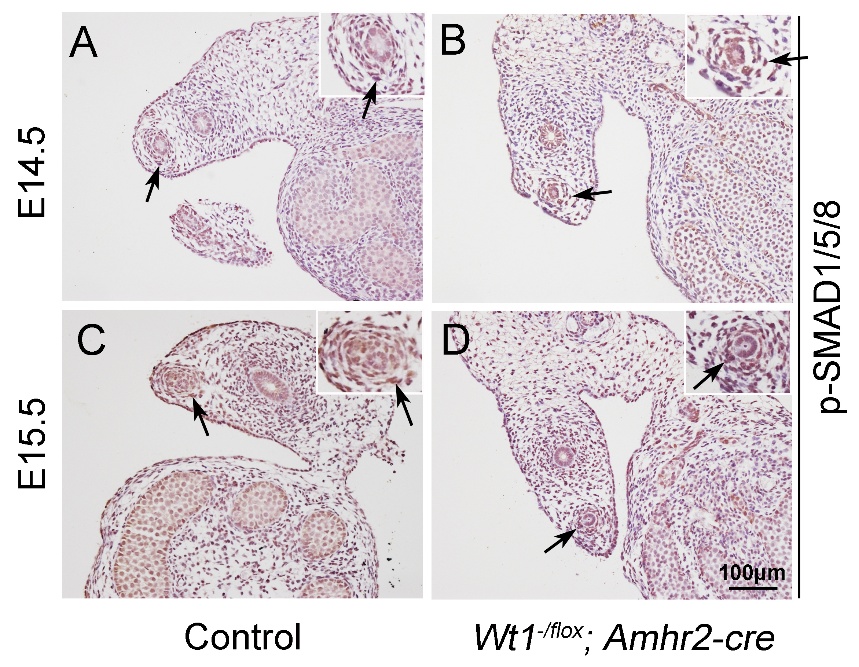


**Fig. S4. Activation of SMAD1/5/8 was unchanged in the Müllerian duct mesenchyme of *Wt1^-/flox^; Amhr2-cre* male mice.** The expression of phosphorylated SMAD1/5/8 (arrows) in Müllerian duct mesenchyme from control and *Wt1^-/flox^; Amhr2-cre* male mice at E14.5 and E15.5 was examined by immunohistochemical staining.


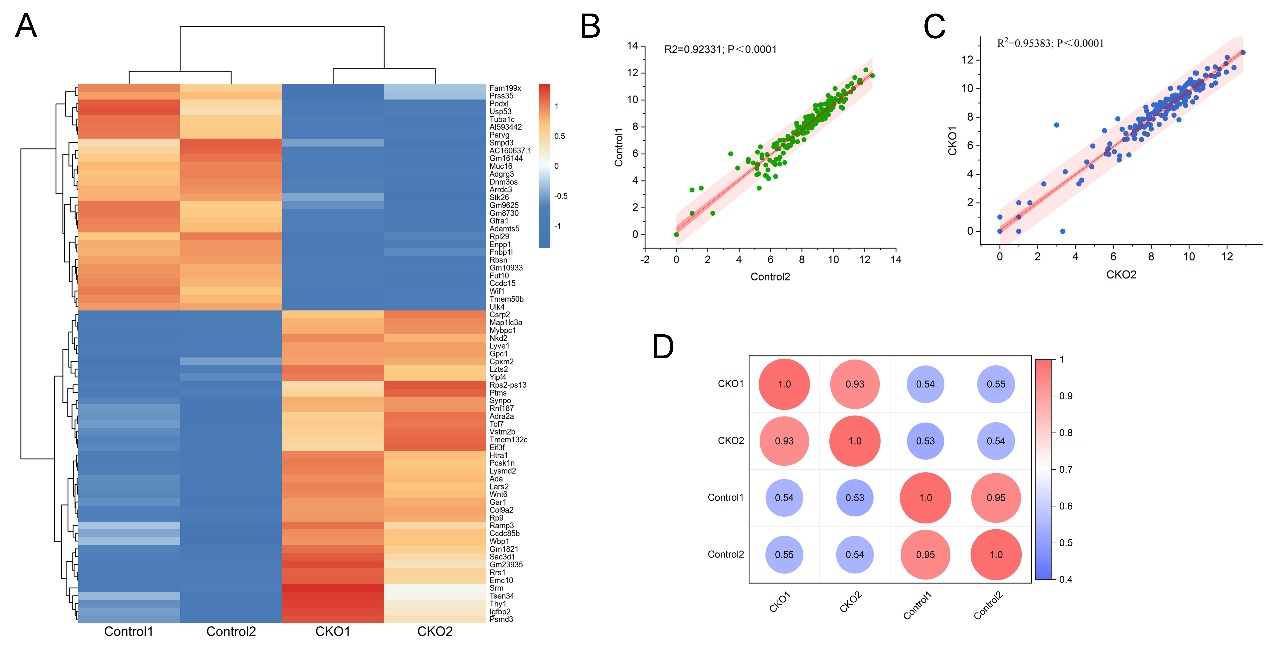


**Fig. S5. RNA-seq analysis of GFP-positive Müllerian duct mesenchymal cells from control and *Wt1^-/flox^; Amhr2-cre; mTmG* males.** A. Heatmap of differentially expressed genes between *Wt1^+/flox^; Amhr2-cre; mTmG* (Control) and *Wt1^-/flox^; Amhr2-cre; mTmG* (CKO) male mice at E14.5. B-C. Pearson correlation analysis of transcriptomes for control (B, green dots) and *Wt1^-/flox^; Amhr2-cre; mTmG* groups (C, blue dots). Each dot represents a single gene. Red line, linear best fit; pink band, 95% confidence interval. The high R² values (Control: 0.9233; CKO: 0.9538; P < 0.0001) confirm robust reproducibility within groups. D. Sample correlation heatmap. Red and larger circles indicate higher correlation; blue and smaller circles indicate lower correlation.


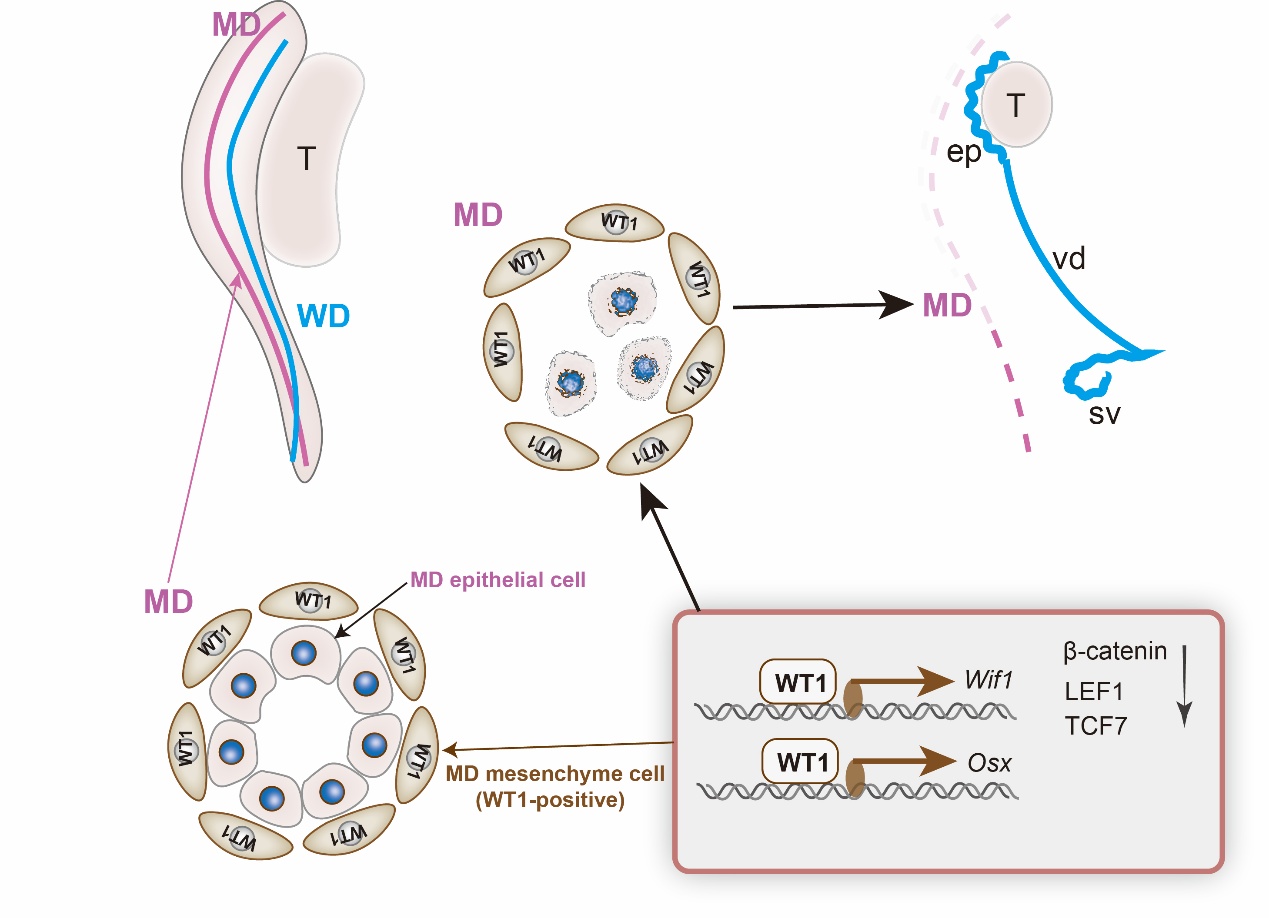


**Fig. S6. Schematic representation of mesenchymal WT1-mediated** **Müllerian duct regression in male mice.** During early embryonic development, the Müllerian duct (MD) and Wolffian duct (WD) are both present within the mesonephroi of male and female mice. After sex determination, the MD regresses in males, whereas the WD differentiates into the male reproductive tract, including the seminal vesicles (sv), vas deferens (vd), and epididymis (ep). MD regression is initiated by AMH secreted from Sertoli cells of the testis (T), which acts through its receptors in the MD mesenchyme. *Wt1* expressed in the MD mesenchyme promotes MD regression by inducing *Wif1* transcription, thereby suppressing β-catenin signaling and reducing the expression of its binding partners LEF1 and TCF7. In addition, *Osx* is another WT1 target gene in the mesenchyme whose upregulation may further contribute to MD regression. Mesenchyme-specific inactivation of *Wt1* results in MD retention in male mice without markedly affecting the expression of AMH or its receptors.

**Table S1. Primers for real-time PCR.**

| Gene Symbol | RT Forward Primer 5’ to 3’ | RT Reverse Primer 5’ to 3’ |
| --- | --- | --- |
| *Amh* | TGGTGCTAACCGTGGACTTC | AGCCAAATAGAAAGGCTTGCA |
| *Amhr2* | GCAGCACAAGTATCCCCAAAC | GTCTCGGCATCCTTGCATCTC |
| *Acvr1* | ATGGTCGATGGAGTAATGATCCT | TGCTCATAAACCTGAAAGCAGC |
| *Bmpr1a* | TGCAAGGATTCACCGAAAGC | TGCCATCAAAGAACGGACCTAT |
| *Wif1* | GATCCAACTGTCAATGTCCCTT | ACACGGGAAACCAACTTGAAC |
| *Wnt5a* | CCGGGAGGGCGAGCTGTCTACC | TGTCCTACGGCCTGCTTCATTGTTGT |
| *Lef1* | GGCGCCGGCGTCCTTCTAA | CATCCCGGCGGCTGTGTAATCT |
| *Tcf7*  *Osx* | GCGCGGGATAACTACGGAAAGAAG  ATGGCGTCCTCTCTGCTTG | GTGGGGAAGGAGGGCAACAGAAG  TGAAAGGTCAGCGTATGGCTT |
